# Immune Checkpoint Inhibitors for Advanced Melanoma: Experience at a Single Institution in Taiwan

**DOI:** 10.3389/fonc.2020.00905

**Published:** 2020-06-04

**Authors:** Chiao-En Wu, Chan-Keng Yang, Meng-Ting Peng, Pei-Wei Huang, Yu-Fen Lin, Chi-Yuan Cheng, Yao-Yu Chang, Huan-Wu Chen, Jia-Juan Hsieh, John Wen-Cheng Chang

**Affiliations:** ^1^Division of Hematology-Oncology, Department of Internal Medicine, Chang Gung Memorial Hospital at Linkou, Chang Gung University College of Medicine, Taoyuan, Taiwan; ^2^Immuno-Oncology Center of Excellence, Chang Gung Memorial Hospital at Linkou, Taoyuan, Taiwan; ^3^Department of Nursing, Chang Gung Memorial Hospital at Linkou, Taoyuan, Taiwan; ^4^Department of Pharmacy, Chang Gung Memorial Hospital at Linkou, Chang Gung University College of Medicine, Taoyuan, Taiwan; ^5^Department of Dermatology, Chang Gung Memorial Hospital at Linkou, Chang Gung University College of Medicine, Taoyuan, Taiwan; ^6^Department of Medical Imaging and Intervention, Chang Gung Memorial Hospital at Linkou, Chang Gung University College of Medicine, Taoyuan, Taiwan

**Keywords:** melanoma, immunotherapy, nivolumab, ipilimumab, pembrolizumab, immune checkpoint inhibitors

## Abstract

**Background:** Immune checkpoint inhibitors (ICIs) have significantly changed the current approach to cancer treatment. Although the use of ICIs has become the standard of care for advanced melanoma, reports of ICI use among Asian populations with melanoma are limited. Therefore, we conducted this retrospective study to assess the efficacy and safety of ICI use in Taiwanese patients.

**Patients:** Patients with histologically confirmed melanoma treated with ICIs at Linkou Chang Gung Memorial Hospital from January 2014 to July 2019 were retrospectively reviewed. Univariant and multivariant analyses were performed to identify possible prognostic factors.

**Results:** Among 80 patients, 45 were treatment-naïve (56.3%), and 35 received prior systemic drugs other than ICIs. Regarding treatment regimens, patients were treated with ipilimumab (*n* = 9), nivolumab (*n* = 33), pembrolizumab (*n* = 16), or combination drugs (*n* = 22). Nine patients achieved either a complete (*n* = 2) or partial (*n* = 7) response and 13 patients were stable, with a resulting response rate of 11.3% and disease control rate of 27.5%. As of the last follow-up in January 2020, patients treated with combination drugs had longer median progression-free survival (PFS) of 5.6 (95% confidence interval [CI]: 1.6–9.6) months than nivolumab (2.9 months, 95% CI: 1.9–3.9 months), pembrolizumab (3.2 months, 95% CI: 2.6–3.8 months), and ipilimumab (2.6 months, 95% CI: 2.4–2.8 months; *p* = 0.011). No significant differences in overall survival (OS) among the four regimens (*p* = 0.891) were noted. In the multivariate analysis, combination treatment, disease control, and performance ≤ 1 were independent prognostic factors for PFS. Liver metastases and no disease control were independent unfavorable prognostic factors for OS. The most common factor was skin toxicity (45%), followed by endocrine toxicity (18.8%). Patients undergoing combination treatment experienced more frequent and serious adverse events than patients undergoing monotherapy.

**Conclusion:** ICIs demonstrated efficacy and safety in Taiwanese patients with melanoma. Combination treatment showed the greatest efficacy, but this was also accompanied by greater toxicity among the four regimens. In addition, we identified important prognostic factors, such as liver metastases, performance status, and tumor response, for both PFS and OS. These findings could provide physicians with more information to justify clinical outcomes observed in Asian patients with advanced melanoma.

## Introduction

Immune checkpoint inhibitors (ICIs) aim to target the interaction between cancer and immune cells, thus enhancing immunity against tumors. Currently, the availability of ICIs, such as anti-cytotoxic T lymphocyte antigen 4 (CTLA-4) and anti-programmed cell death 1(PD-1)/programmed cell death-ligand 1 monoclonal antibodies, has significantly changed the approach to cancer treatment. Ipilimumab, an anti-CTLA-4 monoclonal antibody, was the first approved ICI that showed efficacy in advanced/metastatic melanoma (1). Subsequently, nivolumab (2) and pembrolizumab (3), both of which are anti-PD-1 antibodies, were approved for advanced melanoma treatment. Combination therapy of ipilimumab and nivolumab has demonstrated better efficacy than either nivolumab or ipilimumab alone, particularly in BRAF-mutant melanoma, but with significantly greater adverse events (AEs) as evidenced in the CheckMate 067 trial (2, 4, 5). The CheckMate 511 study demonstrated a significantly lower incidence of treatment-related grade 3–5 AEs with N3I1 (3-mg/kg nivolumab plus 1-mg/kg ipilimumab) vs. N1I3 (1-mg/kg nivolumab plus 3-mg/kg ipilimumab) without compromising efficacy (6); therefore, N3I1 is widely used in clinical practice for patients undergoing combination treatment. However, the aforementioned studies were conducted in Western countries where cases of acral or mucosal melanoma are rare and thus account for a very small proportion of melanomas.

The spectrum of melanoma in Asians is distinct compared with that in Western population, as acral melanoma and mucosal melanoma account for the majority of melanoma cases (7–11). Tumor mutation burden (TMB) is possibly one of the best biomarkers to predict the response to ICIs (12), and has been validated in melanoma (13). However, TMB of acral and mucosal melanoma was not as high as TMB of cutaneous melanoma (14), so the question arises as to whether ICIs exhibit similar efficacy in Asian melanoma as in Western melanoma, particularly for acral and mucosal melanoma. Previous studies have shown conflicting results, with some reporting no differences with cutaneous melanoma (15, 16) and others showing contrary results (17–19).

To address this issue, we retrospectively reviewed patients with melanoma undergoing ICI treatment in a high-volume tertiary-care cancer center in Taiwan, and analyzed possible prognostic factors.

## Materials and Methods

### Patients

All patients with histologically confirmed melanoma treated at the Chang Gung Memorial Hospital (CGMH), Linkou, from 2014 to 2019 were retrospectively reviewed. A total of 80 ICI-naïve patients with advanced melanoma undergoing ICI treatment with either nivolumab, pembrolizumab, ipilimumab, or combination were included in the study.

### Treatment Regimens and Response Evaluation

The treatment regimens consisted of ipilimumab (3 mg/kg every 3 weeks for maximum of four cycles), nivolumab (3 mg/kg every 2 weeks), pembrolizumab, (2 mg/kg every 3 weeks), or combination of ipilimumab and nivolumab/pembrolizumab until disease progression or intolerant toxicities. The dosing schedule of ICIs was adjusted at the physician's discretion according to the patient's clinical status and toxicity to ICIs. Tumor response was evaluated regularly by physical examination, chest X ray, computed tomography scan, or positron emission tomography scan.

### Patient Characteristics and Evaluation of Outcomes

All patients with advanced melanoma treated from 2014 to 2019 were retrospectively reviewed, and ICI-naïve patients undergoing first-time treatment were included in the current study. Patients who received other systemic treatments prior to ICI therapy, such as chemotherapy, targeted therapy, or cytokine therapy, were also included. The last follow-up timepoint included in the study was January 31, 2020. Patient characteristics, including age, sex, Eastern Cooperative Oncology Group (ECOG) performance status, systemic treatment prior to ICIs, stage of melanoma, and tumor involvement of distant metastases, were recorded.

RECIST 1.1 criteria was used to evaluate the best tumor response as complete response (CR), partial response (PR), stable disease (SD), or progressive disease (PD). Patients who experienced rapid deterioration or lacked radiological evaluation data before death were recorded as not assessed (N/A). Objective response rate (ORR) was the sum of CR and PR; disease control rate (DCR) was the sum of CR, PR, and SD. Progression-free survival (PFS) was defined as the length of time from the first day of ICI treatment until the first clinical or radiological evidence of disease progression, death, or latest follow-up timepoint. Overall survival (OS) was defined as the length of time from the first day of ICI treatment until the date of death or last follow-up.

### Statistical Analysis

To assess the differences among the four regimens, Fisher–Freeman–Halton test of independence was used for categorical variables. Kruskal–Wallis test, a non-parametric (distribution-free) test, was used for continuous variables. Survival was estimated using the Kaplan–Meier method and was compared using the log-rank test. Univariate and multivariate analyses were performed to evaluate possible prognostic factors. Only significant prognostic factors from univariate analysis were further analyzed using multivariate analysis. IBM SPSS Statistics for Windows (Version 20.0, Armonk, NY, USA) was used for statistical analyses, where *P* < 0.05 was considered statistically significant. This study was approved by the Institutional Review Board of CGMH (202000182B0). Patient consent to participate was not required because of the retrospective nature of this study, which was approved by the Institutional Review Board of CGMH.

## Results

### Patient Characteristics

A total of 80 patients with advanced ICI-naïve melanoma undergoing ICIs were included in the study. In terms of treatment regimens, patients received ipilimumab (*n* = 9), nivolumab (*n* = 33), pembrolizumab (*n* = 16), or combination (*n* = 22). Among 22 patients undergoing combination treatment, 17 patients received ipilimumab plus nivolumab, and 5 patients received ipilimumab plus pembrolizumab. The median age was 59.6 years, with a range from 22.5 to 82.4 years. Forty patients (50%) were male and 40 patients (50%) were female. Most patients had an ECOG performance status ≤ 1 (*n* = 71, 88.8%). Twenty-seven patients had acral melanoma, 14 patients had cutaneous melanoma, 20 patients had mucosal melanoma, 10 patients had other types of melanoma (including eyes and soft tissue), and 9 patients had unknown primary melanoma. Most patients (*n* = 73, 91.3%) had been diagnosed as stage IV. Lung (*n* = 45) was the most common metastatic site, followed by liver (*n* = 30), bone (*n* = 28), and brain (*n* = 5). Eighteen of 70 patients (25.7%) had a BRAF mutation, and mutation status was unknown in 10 patients.

Except for age, tumor type, and number of metastatic sites, no significant differences of clinical characteristics among different ICI treatment groups were identified. The clinical features and tumor involvement with different regimens are summarized in Table 1.

**Table 1:** Patients' characteristics and association with different regimens.

**Characteristics**	**Regimens**				***P*-value**
	**Ipilimumab (*N* = 9)**	**Nivolumab (*N* = 33)**	**Pembrolizumab (*N* = 16)**	**Combination (*N* = 22)**	
Age, median (IQR)	63 (15)	63 (18)	51.5 (14)	57.5 (31)	0.027
≤60	3 (33.3)	11 (33.3)	12 (75.0)	14 (63.6)	0.016
>60	6 (66.7)	22 (66.7)	4 (25.0)	8 (36.4)	
**Sex**					**0.348**
Male (*n* = 40)	7 (77.8)	15 (45.5)	7 (43.8)	11 (50.0)	
Female (*n* = 40)	2 (22.2)	18 (54.5)	9 (56.3)	11 (50.0)	
**Performance status**					**0.621**
0/1 (*n* = 71)	9 (100.0)	28 (84.8)	14 (87.5)	20 (90.9)	
2/3 (*n* = 9)	0	5 (15.2)	2 (12.5)	2 (9.1)	
**Location**					**0.262**
Four limbs (*n* = 31)	6 (66.7)	13 (39.4)	5 (31.3)	7 (31.8)	
Head and neck (*n* = 18)	0	4 (12.1)	6 (37.5)	8 (36.4)	
Truck (*n* = 22)	2 (22.2)	12 (36.4)	3 (18.8)	5 (22.7)	
Unknown (*n* = 9)	1 (11.1)	4 (12.1)	2 (12.5)	2 (9.1)	
**Type**					**0.024**
Acral (*n* = 27)	6 (66.7)	13 (39.4)	3 (18.8)	5 (22.7)	
Cutaneous (*n* = 14)	0	3 (9.1)	2 (12.5)	9 (40.9)	
Mucosal (*n* = 20)	2 (22.2)	11 (33.3)	5 (31.3)	2 (9.1)	
Others (*n* = 10)	0	2 (6.1)	4 (25.0)	4 (18.2)	
Unknown (*n* = 9)	1 (11.1)	4 (12.1)	2 (12.5)	2 (9.1)	
**Lung metastasis**					**0.074**
No (*n* = 35)	3 (33.3)	18 (54.5)	9 (56.3)	5 (22.7)	
Yes (*n* = 45)	6 (66.7)	15 (45.5)	7 (43.8)	17 (77.3)	
**Liver metastasis**					**0.245**
No (*n* = 50)	8 (88.9)	21 (63.6)	10 (62.5)	11 (50.0)	
Yes (*n* = 30)	1 (11.1)	12 (36.4)	6 (37.5)	11 (50.0)	
**Bone metastasis**					**0.387**
No (*n* = 52)	4 (44.4)	23 (69.7)	12 (75.0)	13 (59.1)	
Yes (*n* = 28)	5 (55.6)	10 (30.3)	4 (25.0)	9 (40.9)	
**Brain metastasis**					**0.925**
No (*n* = 75)	8 (88.9)	31 (93.9)	15 (93.8)	21 (95.5)	
Yes (*n* = 5)	1 (11.1)	2 (6.1)	1 (6.3)	1 (4.5)	
**No. of metastatic sites**					**0.014**
≤1 (*n* = 23)	0	12 (36.4)	8 (50.0)	3 (13.6)	
>1 (*n* = 57)	9 (100.0)	21 (63.6)	8 (50.0)	19 (86.4)	
**Stage**					**0.142**
III (*n* = 7)	0	4 (12.1)	3 (18.8)	0	
IV (*n* = 73)	9 (100.0)	29 (87.9)	13 (81.3)	22 (100.0)	
**BRAF gene mutation**					**0.530**
No (*n* = 52)	7 (77.8)	23 (79.3)	11 (78.6)	11 (61.1)	
Yes (*n* = 18)	2 (22.2)	6 (20.7)	3 (21.4)	7 (38.9)	
**Immunotherapy therapy**					**0.277**
First-line (*n* = 45)	4 (44.4)	19 (57.6)	12 (75.0)	10 (45.5)	
Second-or later-line (*n* = 35)	5 (55.6)	14 (42.4)	4 (25.0)	12 (54.5)	
**Response**					**0.335**
CR/PR (*n* = 9)	0	3 (9.1)	2 (12.5)	4 (18.2)	
SD (*n* = 13)	0	5 (15.2)	2 (12.5)	6 (27.3)	
PD (*n* = 47)	8 (88.9)	22 (66.7)	9 (56.3)	8 (36.4)	
N/A (*n* = 11)	1 (11.1)	3 (9.1)	3 (18.8)	4 (18.2)	

*CR, complete response; PR, partial response; SD, stable disease; PD, progressive disease; N/A, not assessed*.

### Prior Treatments Before ICIs

Forty-five (56.3%) patients were treated with ICIs as a first-line systemic treatment, and 35 (43.7%) patients were treated with these as second- or late-line treatment. The details of prior treatments are summarized in Table 2.

**Table 2:** Prior systemic treatment before different regimens.

	**Ipilimumab (*****N*** **=** **9)**		**Nivolumab (*****N*** **=** **29)**		**Pembrolizumab (*****N*** **=** **15)**		**Combination (*****N*** **=** **18)**	
	***n***	**%**	***n***	**%**	***n***	**%**	***n***	**%**
Any treatment	5	55.6%	14	48.3%	4	26.7%	12	66.7%
BRAFi ± MEKi	0	0.0%	3	10.3%	3	20.0%	4	22.2%
Cytokines	3	33.3%	10	34.5%	1	6.7%	7	38.9%
Chemotherapy	5	55.6%	10	34.5%	0	0.0%	10	55.6%

*BRAFi, BRAF inhibitor; MEKi, MEK inhibitor*.

### Efficacy of ICIs With Different Regimens

**Figure 1:**
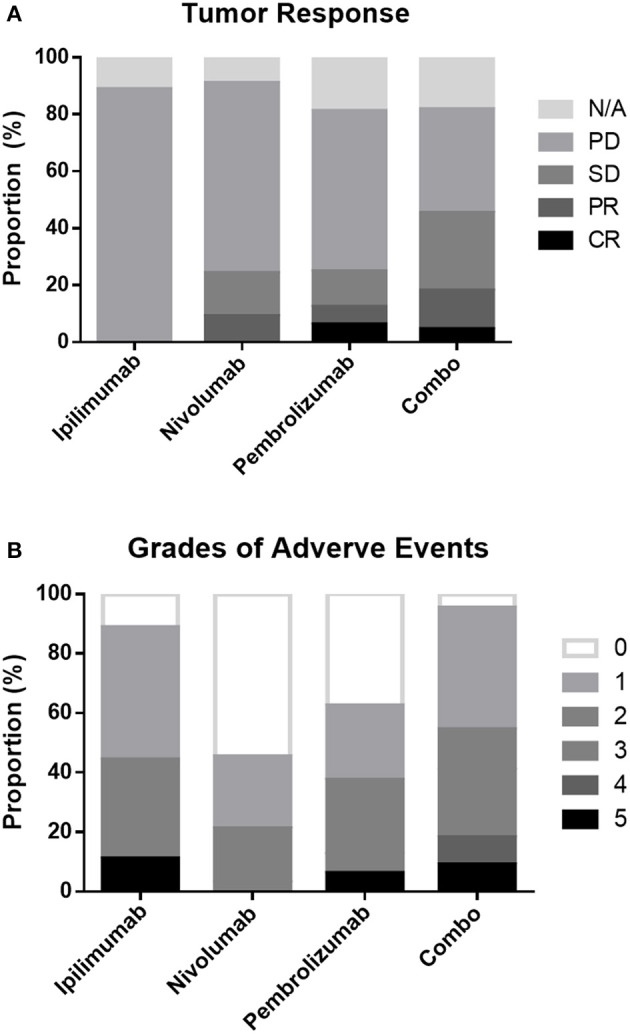
Proportion of tumor responses **(A)** and adverse events **(B)** following different treatment regimens.

Among the patients with an evaluable response, 2 patients achieved CR, 7 achieved PR, 13 achieved SD, and 47 had PD as their best response. Eleven patients had no response evaluation data, and most experienced rapid progression without radiological confirmation. The ORR and DCR were 11.3 and 27.5% in the entire cohort and 12.7 and 31.0% in evaluable patients, respectively. Patients treated with combination drugs had a numerically but non-significantly higher ORR and DCR (Table 1, Figure 1A).

**Figure 2:**
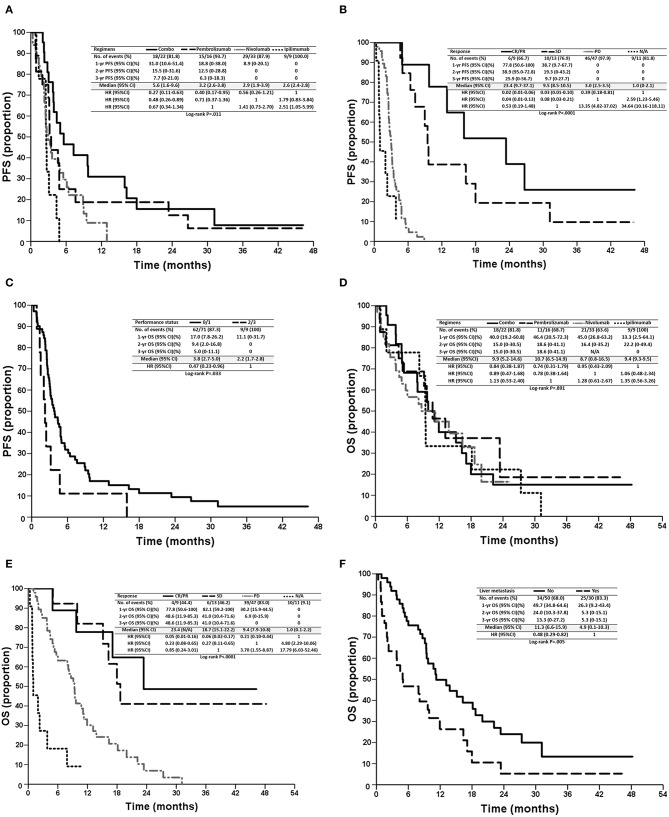
Kaplan–Meier survival curves of PFS **(A–C)** and OS **(D–F)** of patients, stratified according to prognostic factors, regimens **(A,D)**, tumor responses **(B,E)**, performance status **(E)**, and liver metastasis **(F)**. PFS, progression-free survival; OS, overall survival; CR, complete response; PR, partial response; SD, stable disease; PD, progressive disease; N/A, not assessed.

Patients in the combination treatment group had a longer PFS of 5.6 (95% CI: 1.6–9.6) months than that of nivolumab (2.9 months, 95% CI: 1.9–3.9 months), pembrolizumab (3.2 months, 95% CI: 2.6–3.8 months), and ipilimumab (2.6 months, 95% CI: 2.4–2.8 months) treatment groups (*p* = 0.011, Table 2, Figure 2A). However, there were no significant differences in OS observed among the four regimens (*p* = 0.891; Figure 2D), possibly because most patients received sequential systemic treatment after progression (Table 3).

**Table 3:** Subsequent systemic treatment after different regimens.

	**Ipilimumab (*****N*** **=** **9)**		**Nivolumab (*****N*** **=** **29)**		**Pembrolizumab (*****N*** **=** **15)**		**Combination (*****N*** **=** **18)**	
	***n***	**%**	***n***	**%**	***n***	**%**	***n***	**%**
Any treatment	8	88.9%	20	69.0%	8	53.3%	7	38.9%
Anti-PD-1	7	77.8%	5	17.2%	1	6.7%	4	22.2%
Ipilimumab	0	0.0%	11	37.9%	4	26.7%	4	22.2%
BRAFi ± MEKi	1	11.1%	1	3.4%	0	0.0%	4	22.2%
Other Targeted Therapy	0	0.0%	2	6.9%	0	0.0%	0	0.0%
Cytokines	0	0.0%	8	27.6%	2	13.3%	1	5.6%
Chemotherapy	0	0.0%	4	13.8%	3	20.0%	1	5.6%

*PD-1, programmed cell death 1; BRAFi, BRAF inhibitor; MEKi, MEK inhibitor*.

### Subsequent Systemic Treatment After ICIs

Forty-three (53.8%) patients received systemic treatment after ICIs, including pembrolizumab (*n* = 8, 53.3%), nivolumab (*n* = 20, 69.0%), ipilimumab (*n* = 8, 88.9%), and combination (*n* = 7, 38.9%). The details of systemic treatments are summarized in Table 3.

### Identification of Prognostic Factors for PFS

In the univariate analysis, sex (*p* = 0.014), performance status (*p* = 0.033), treatment regimens (*p* = 0.011), and tumor response (*p* < 0.001) were significant prognostic factors of PFS. In the multivariate analysis, combination treatment [vs. ipilimumab, hazards ratio (HR): 0.31, 95% CI: 0.12–0.81, *p* = 0.017, Figure 2A], tumor responses with CR/PR (vs. PD, HR: 0.05, 95% CI: 0.02–0.19, *p* < 0.0001), SD (vs. PD, HR: 0.11, 95% CI: 0.04–0.30, *p* < 0.0001), N/A (vs. PD, HR: 5.13, 95% CI: 2.08–12.66, *p* < 0.001, Figure 2B), and a performance status ≤ 1 (vs. > 2, HR: 0.42, 95% CI: 0.20–0.89, *p* = 0.024, Figure 2C) were independent favorable prognostic factors for PFS (Table 4). In addition, pembrolizumab showed a tendency for longer PFS than ipilimumab (HR: 0.46, 95% CI: 0.19–1.13, *p* = 0.090), and nivolumab had numerically longer PFS than ipilimumab (HR: 0.67, 95% CI: 0.30–1.51, *p* = 0.337).

**Table 4:** Univariate and multivariate analysis of prognostic factors in progression-free survival.

**Parameters**	**Median (months)**	**95% CI of median**	***P*-value**	**Hazard ratio**	**95% CI of HR**	***P*-value**
**Age**			**0.667**	**–**		
≤60 (*n* = 40)	3.5	2.6–4.3				
>60 (*n* = 40)	3.3	1.2–5.3				
**Sex**			**0.014**			
Male (*n* = 40)	4.8	2.9–6.7		0.73	0.44–1.21	.219
Female (*n* = 40)	3.1	2.5–3.7		1		
**Performance status**			**0.033**			
0/1 (*n* = 71)	3.8	2.7–5.0		0.42	0.20–0.89	.024
2/3 (*n* = 9)	2.2	1.7–2.8		1		
**Location**			**0.439**	**–**		
Four limbs (*n* = 31)	2.7	2.5–2.9				
Head and neck (*n* = 18)	4.4	1.9–7.0				
Truck (*n* = 22)	3.1	1.9–4.3				
Unknown (*n* = 9)	5.0	3.2–6.8				
**Type**			**0.488**	**–**		
Acral (*n* = 27)	2.6	2.4–2.8				
Cutaneous (*n* = 14)	4.8	3.6–6.1				
Mucosal (*n* = 20)	3.1	2.7–3.4				
Others (*n* = 10)	3.1	0.1–6.8				
Unknown (*n* = 9)	5.0	3.2–6.8				
**Lung metastasis**			**0.809**	**–**		
No (*n* = 35)	3.6	2.4–4.9				
Yes (*n* = 45)	3.2	2.1–4.3				
**Liver metastasis**			**0.185**	**–**		
No (*n* = 50)	3.7	2.4–5.1				
Yes (*n* = 30)	3.0	2.1–3.9				
**Bone metastasis**			**0.368**	**–**		
No (*n* = 52)	3.5	1.8–5.1				
Yes (*n* = 28)	3.2	1.5–4.9				
**Brain metastasis**			**0.796**	**–**		
No (*n* = 75)	3.6	2.8–4.4				
Yes (*n* = 5)	2.9	2.3–3.6				
**No. of metastatic sites**			**0.686**	**–**		
≤1(*n* = 23)	3.3	2.7–3.8				
>1 (*n* = 57)	3.7	2.8–4.7				
**Stage**			**0.654**	**–**		
III (*n* = 7)	4.9	4.7–5.2				
IV (*n* = 73)	3.4	2.7–4.2				
**BRAF gene mutation**			**0.205**	**–**		
No (*n* = 52)	3.4	1.9–5.0				
Yes (*n* = 18)	3.8	2.0–5.7				
**Immunotherapy therapy**			**0.267**	**–**		
First-line (*n* = 45)	4.7	3.2–6.2				
Second-or later-line (*n* = 35)	2.7	2.1–3.3				
**Regimens**			**0.011**			
Ipilimumab (*n* = 9)	2.6	2.4–2.8		1		
Nivolumab (*n* = 33)	2.9	1.9–3.9		0.67	0.30–1.51	0.337
Pembrolizumab (*n* = 16)	3.2	2.6–3.8		0.46	0.19–1.13	0.090
Combination (*n* = 22)	5.6	1.6–9.6		0.31	0.12–0.81	0.017
**Response**			**<0.0001**			
CR/PR (*n* = 9)	23.4	9.7–37.1		0.05	0.02–0.19	<0.0001
SD (*n* = 13)	9.5	8.5–10.5		0.11	0.04–0.30	<0.0001
PD (*n* = 47)	3.0	2.5–3.5		1		
N/A (*n* = 11)	1.0	0.1–2.1		5.13	2.08–12.66	<0.001

*CI, confidence interval; CR, complete response; PR, partial response; SD, stable disease; PD, progressive disease; N/A, not assessed*.

### Identification of Prognostic Factors for OS

In the univariate analysis, liver metastasis (*p* = 0.005), performance status (*p* = 0.041), and tumor response (*p* < 0.001) were significant prognostic factors for OS. In the multivariate analysis, tumor responses with CR/PR (vs. PD, HR: 0.21, 95% CI: 0.07–0.60, *p* = 0.004), SD (vs. PD, HR: 0.30, 95% CI: 0.13–0.73, *p* = 0.008), N/A (vs. PD, HR: 4.99, 95% CI: 2.32–10.74, *p* < 0.001, Figure 2E), and no liver metastasis (vs. liver metastasis, HR: 0.51, 95% CI: 0.28–0.91, *p* = 0.022, Figure 2F) were independent favorable prognostic factors for OS (Table 5). Among 30 patients with liver metastasis, 3 patients had PR and 4 patients had SD resulting in ORR of 10% and DCR of 23.3% which were not statistically different from the patients without liver metastasis.

**Table 5:** Univariate and multivariate analysis of prognostic factors in overall survival.

**Parameters**	**Median (months)**	**95% CI of median**	***P*-value**	**Hazard ratio**	**95% CI of HR**	***P*-value**
**Age**			**0.977**	**–**		
≤60 (*n* = 40)	11.1	8.8–13.4				
>60 (*n* = 40)	9.4	7.0–11.7				
**Sex**			**0.190**	**–**		
Male (*n* = 40)	10.7	8.5–12.9				
Female (*n* = 40)	9.7	8.4–11.0				
**Performance status**			**0.041**			
0/1 (*n* = 71)	10.7	8.4–13.1		0.75	0.33–1.70	0.495
2/3 (*n* = 9)	5.7	0.1–16.3		1		
**Location**			**0.333**	**–**		
Four limbs (*n* = 31)	9.5	5.1–13.9				
Head and neck (*n* = 18)	11.1	8.7–13.5				
Truck (*n* = 22)	9.1	3.6–14.7				
Unknown (*n* = 9)	18.7	1.7–35.7				
**Type**			**0.236**	**–**		
Acral (*n* = 27)	10.7	6.1–15.4				
Cutaneous (*n* = 14)	11.1	0.6–21.6				
Mucosal (*n* = 20)	9.4	5.1–13.6				
Others (*n* = 10)	4.9	0.1–12.2				
Unknown (*n* = 9)	18.7	1.7–35.7				
**Lung metastasis**			**0.539**	**–**		
No (*n* = 35)	10.7	5.3–16.1				
Yes (*n* = 45)	9.7	7.4–12.0				
**Liver metastasis**			**0.005**			
No (*n* = 50)	11.3	6.6–15.9		0.51	0.28–0.91	0.022
Yes (*n* = 30)	4.9	0.1–10.3		1		
**Bone metastasis**			**0.182**	**–**		
No (*n* = 52)	11.3	5.2–17.4				
Yes (*n* = 28)	8.7	6.8–10.5				
**Brain metastasis**			**0.303**	**–**		
No (*n* = 75)	9.7	7.9–11.5				
Yes (*n* = 5)	31.1	N/A				
**No. of metastatic sites**			**0.474**			
≤1(*n* = 23)	10.7	8.2–13.3				
>1 (*n* = 57)	9.5	6.0–12.9				
**Stage**			**0.513**	**–**		
III (*n* = 7)	13.1	6.1–20.2				
IV (*n* = 73)	9.7	7.6–11.7				
**BRAF gene mutation**			**0.240**	**–**		
No (*n* = 52)	11.1	6.9–15.3				
Yes (*n* = 18)	7.9	0.1–16.8				
**Immunotherapy therapy**			**0.088**	**–**		
First-line (*n* = 45)	11.3	7.1–15.5				
Second-or later-line (*n* = 35)	7.9	4.3–11.6				
**Regimens**			**0.891**	**–**		
Ipilimumab (*n* = 9)	9.4	9.3–9.5				
Nivolumab (*n* = 33)	8.7	0.8–16.5				
Pembrolizumab (*n* = 16)	10.7	6.5–14.9				
Combination (*n* = 22)	9.9	5.2–14.6				
**Response**			**<0.0001**			
CR/PR (*n* = 9)	23.4	N/A		0.21	0.07–0.60	0.004
SD (*n* = 13)	18.7	15.1–22.2		0.30	0.13–0.73	0.008
PD (*n* = 47)	9.4	7.9–10.8		1		
N/A (*n* = 11)	1.0	0.1–2.2		4.99	2.32–10.74	<0.0001

*CI, confidence interval; CR, complete response; PR, partial response; SD, stable disease; PD, progressive disease; N/A, not assessed*.

### AEs

Overall, patients treated with combination treatment had the most frequent AEs across all grades (*p* < 0.001) and more grade 3 AEs (*p* = 0.002) than other ICIs treatment. Grade 3–5 treatment-related AEs occurred in 11.1, 3, 12.5, and 40.9% of patients in the ipilimumab, nivolumab, pembrolizumab, and combination groups, respectively. Skin-related AEs were the most common, followed by endocrine and pulmonary AEs. Patients treated with combination displayed a trend for skin, endocrine, and lung related AEs, although these results did not reach statistical significance. The details of all grades of AE are summarized in Table 6 and Figure 1B.

**Table 6:** Adverse events in the as-treated population.

**Adverse events**	**Regimens**				***P*-value**
	**Ipilimumab (*N* = 9)**	**Nivolumab (*N* = 33)**	**Pembrolizumab (*N* = 16)**	**Combo (*N* = 22)**	
**Skin**					
Any grade (*n* = 36)	4 (44.4)	10 (30.3)	7 (43.8)	15 (68.2)	0.054
Grade 3–5 (*n* = 2)	0	1 (3.0)	0	1 (4.5)	>0.999
**Colitis**					
Any grade (*n* = 10)	2 (22.2)	4 (12.1)	1 (6.3)	3 (13.6)	0.710
Grade 3–5 (*n* = 1)	0	0	0	1 (4.5)	0.588
**Liver**					
Any grade (*n* = 7)	1 (11.1)	1 (3.0)	1 (6.3)	4 (18.2)	0.242
Grade 3–5 (*n* = 4)	1 (11.1)	0	0	3 (13.6)	0.045
**Neurology**					
Any grade (*n* = 2)	0	0	0	2 (9.1)	0.231
Grade 3–5 (*n* = 1)	0	0	0	1 (1.3)	0.588
**Lung**					
Any grade (*n* = 8)	0	1 (3.0)	2 (12.5)	5 (22.7)	0.067
Grade 3–5 (*n* = 6)	0	0	2 (12.5)	4 (18.2)	0.032
**Endocrine**					
Any grade (*n* = 15)	1 (11.1)	4 (12.5)	3 (18.8)	7 (31.8)	0.308
Grade 3–5 (*n* = 2)	0	0	0	2 (9.1)	0.231
**Heart**					
Any grade (*n* = 1)	0	0	0	1 (4.5)	0.588
Grade 3–5 (*n* = 1)	0	0	0	1 (4.5)	0.588
**Fatigue**					
Any grade (*n* = 11)	3 (33.3)	3 (9.1)	1 (6.3)	4 (18.2)	0.206
Grade 3–5 (*n* = 0)	0	0	0	0	–
**Vitiligo**					
Any grade (*n* = 9)	1 (11.1)	3 (9.1)	1 (6.3)	4 (18.2)	0.730
Grade 3–5 (*n* = 0)	0	0	0	0	–
**Overall**					
Any grade (*n* = 54)	8 (88.9)	15 (45.5)	10 (62.5)	21 (95.5)	<0.001
Grade 3–5 (*n* = 13)	1 (11.1)	1 (3.0)	2 (12.5)	9 (40.9)	0.002

## Discussion

We retrospectively reviewed ICI-naïve patients with melanoma undergoing various ICI treatments in Taiwan. Overall, the ORR was 11.3%, and DCR was 27.5%. Combination treatment with anti-CTLA-4 and anti-PD-1 antibodies provided numerically higher ORR and DCR, but this result was not statistically significant. In addition, combination treatment was associated with similar OS but significantly longer PFS with a greater risk of AEs than monotreatment, possibly because of subsequent treatments. In addition, we identified important prognostic factors, including liver metastases, performance status, and tumor response, for both PFS and OS. These findings could provide physicians with more information to justify clinical outcomes in patients with advanced melanoma in acral/mucosal-melanoma-predominant areas.

Combination treatment demonstrated the greatest ORR of 18.2% and DCR of 45.5%, and anti-PD-1 (nivolumab or pembrolizumab) showed an ORR of ~10% and DCR of ~25%. Both ORR and DCR were lower than those in previous prospective studies (4, 6, 20–24). In the CheckMate 067 study, the ORR and DCR were 48 and 70%, respectively, in the N3I1 group, 44 and 54%, respectively, in the nivolumab group, and 19 and 41%, respectively, in the ipilimumab group (4). Additionally, In three clinical trials for advanced melanoma (KEYNOTE-001, KEYNOTE-002, and KEYNOTE-006), pembrolizumab provided ORRs of 30–40% in previously treated and treatment-naïve patients (6, 20–24). The low ORR and DCR seen in the current study may be a result of a high proportion of acral/mucosal melanoma with lower TMB than cutaneous melanoma (14). However, the response of ICIs in cutaneous melanoma was not as good as the results reported in clinical trials. The genetic alterations such as copy number variations of CDK4 pathway-related genes differ in Asian and Western melanoma which may contribute low response in Asian melanoma (25, 26). These findings support our hypothesis that ICIs function differently in Asian patients with melanoma than in Western patients with melanoma. Further studies are needed to explore possible mechanism and investigate novel treatment to improve the efficacy of ICIs in Asian melanoma particularly for acral and mucosal melanomas.

Furthermore, median PFS for acral melanoma (2.6 months) and mucosal melanoma (3.1 months) was shorter than that for cutaneous melanoma (4.8 months), indicating that tumor histology plays some sort of role in response to ICIs (Table 4). In a retrospective study of 60 individuals with acral (*n* = 25)/mucosal (*n* = 35) melanoma treated with anti-PD-1 (either nivolumab or pembrolizumab), ORR was 32% and median PFS was 4.1 months in patients with acral melanomas and ORR was 23% and median PFS was 3.9 months in patients with mucosal melanomas (15). Although the authors concluded that the ORR was comparable to published rates in cutaneous melanoma, the numerically lower ORR and shorter PFS of patients with acral/mucosal melanoma should be concerning in such subtypes of melanoma. Similar findings were reported in various melanoma studies, including a phase II study of Japanese patients treated with nivolumab (27), another phase II study of 30 Japanese patients treated with N1I3 combination (28), an observational study of 124 Japanese patients treated with nivolumab (29), and a phase 1b study (Keynote 151) of 103 Chinese melanoma patients treated with pembrolizumab as a second-line therapy (30). The *post-hoc* analysis of KEYNOTE 001, 002, and 006 trials reported pembrolizumab use in 84 patients with advance mucosal melanoma, with an ORR of 19% (compared with 33% in patients with a cutaneous melanoma), a DCR of 31%, a median PFS of 2.8 months, and a median OS of 11.3 months, which were inferior than those reported for patients with non-mucosal melanoma, thus supporting previous findings (17).

Combination treatment lead to a significantly higher frequency of any grade (95.5%) and grade ≥ 3 (40.9%) AEs than monotherapy, which was comparable with AE rates reported in previous phase III (2, 4–6) and phase II (28) studies. Unfortunately, four patients experienced AE-related death in the current study, and all occurred in the early stages of ICI treatment (prior to 2016). Presently, with a combination of comprehensive understanding, early reorganization and diagnosis, and adequate management of immune-related AEs (irAEs), most irAEs can be diagnosed and treated in early stages (6). Thus, there were no more deaths from AEs since 2017 in the current series. Recent studies found the occurrence of AEs might impact the therapeutic efficacy of ICIs. Yamazaki et al. (29) reported that the occurrence of skin-related and endocrine-related irAEs had a significant impact on PFS of patients with melanoma treated with nivolumab. Moreover, Fujisawa et al. (31) demonstrated that occurrences of endocrine-related irAEs were associated with longer OS of patients treated with ipilimumab after nivolumab. Interestingly, the development of vitiligo was correlated with better responses to ICIs, particularly in patients with melanoma, possibly because both melanocytes and melanomas share common antigens that are recognized by the activated immune response (32). Therefore, it is critical to manage the occurrence of irAEs appropriately, as patients with particular irAEs experience better survival.

The current retrospective analysis has some limitations. The nature of a retrospective study always involves biases. The limited number of patients with melanoma and imbalanced characteristics among different ICIs were the major limitations owing to a low prevalence of melanoma in the area. This made it difficult to do further subgroup analysis such as the influence of different regimens in subtypes of melanoma. Indeed, the economic burden to utilize ICIs as a therapeutic option has made this therapy unaffordable for most patients until recently, as reimbursement by national health insurance in Taiwan has been made available since April 2019. Upon consideration of all prognostic factors investigated for PFS and OS, we did not include few factors such as LDH and CRP reported by previous studies as these were not available for some patients before ICI administration. Furthermore, these patients were treated in a single, high-volume tertiary-care institute, which could not fully capture the type of real-world practice observed in smaller, peripheral clinics. However, the homogeneity of standardized treatment by medical oncologists in such a cancer institute could attenuate the weight of confounding factors.

In conclusion, the current study demonstrated clinical experience of ICI use in Taiwanese patients with melanoma. To our knowledge, this is the first report to compare different ICIs, including anti-PD-1, anti-CTLA-4, and combination treatment, in Asian population. Although ICIs were less efficacious in Taiwanese patients with melanoma, ICIs still provide an alternative option for Taiwanese patients seeking a robust response profile with tolerable toxicity.

## Data Availability Statement

The raw data supporting the conclusions of this article will be made available by the authors, without undue reservation, to any qualified researcher.

## Ethics Statement

The studies involving human participants were reviewed and approved by Institutional Review Board of CGMH (202000182B0). Written informed consent for participation was not required for this study in accordance with the national legislation and the institutional requirements.

## Author Contributions

C-EW, C-KY, M-TP, P-WH, C-YC, and JC contributed conception and design of the study. C-EW, Y-FL, and JC organized the database. C-EW, C-YC, Y-YC, H-WC, and JH performed the statistical analysis. C-EW wrote the first draft of the manuscript. C-EW, Y-YC, H-WC, and C-EW wrote sections of the manuscript. All authors contributed to manuscript revision, read, and approved the submitted version.

## Conflict of Interest

The authors declare that the research was conducted in the absence of any commercial or financial relationships that could be construed as a potential conflict of interest.
